# Multi-Country analysis of palm oil consumption and cardiovascular disease mortality for countries at different stages of economic development: 1980-1997

**DOI:** 10.1186/1744-8603-7-45

**Published:** 2011-12-16

**Authors:** Brian K Chen, Benjamin Seligman, John W Farquhar, Jeremy D Goldhaber-Fiebert

**Affiliations:** 1 Asia Health Policy Program and Center for East Asian Studies, Shorenstein Asia-Pacific Research Center, 616 Serra Street E301, Stanford University, Stanford, CA, USA; 2 Stanford University School of Medicine, Stanford University, 291 Campus Drive, Room LK3C02, Li Ka Shing Building, 3rd Floor, Stanford, CA, USA; 3 Stanford Prevention Research Center, Stanford University School of Medicine, 1265 Welch Road, Medical School Office Building (MC 5411), CA, USA; 4 Stanford Health Policy, Centers for Health Policy and Primary Care and Outcomes Research, Stanford University, 117 Encina Commons, Stanford, CA, USA

## Abstract

**Background:**

Cardiovascular diseases represent an increasing share of the global disease burden. There is concern that increased consumption of palm oil could exacerbate mortality from ischemic heart disease (IHD) and stroke, particularly in developing countries where it represents a major nutritional source of saturated fat.

**Methods:**

The study analyzed country-level data from 1980-1997 derived from the World Health Organization's Mortality Database, U.S. Department of Agriculture international estimates, and the World Bank (234 annual observations; 23 countries). Outcomes included mortality from IHD and stroke for adults aged 50 and older. Predictors included per-capita consumption of palm oil and cigarettes and per-capita Gross Domestic Product as well as time trends and an interaction between palm oil consumption and country economic development level. Analyses examined changes in country-level outcomes over time employing linear panel regressions with country-level fixed effects, population weighting, and robust standard errors clustered by country. Sensitivity analyses included further adjustment for other major dietary sources of saturated fat.

**Results:**

In developing countries, for every additional kilogram of palm oil consumed per-capita annually, IHD mortality rates increased by 68 deaths per 100,000 (95% CI [21-115]), whereas, in similar settings, stroke mortality rates increased by 19 deaths per 100,000 (95% CI [-12-49]) but were not significant. For historically high-income countries, changes in IHD and stroke mortality rates from palm oil consumption were smaller (IHD: 17 deaths per 100,000 (95% CI [5.3-29]); stroke: 5.1 deaths per 100,000 (95% CI [-1.2-11.0])). Inclusion of other major saturated fat sources including beef, pork, chicken, coconut oil, milk cheese, and butter did not substantially change the differentially higher relationship between palm oil and IHD mortality in developing countries.

**Conclusions:**

Increased palm oil consumption is related to higher IHD mortality rates in developing countries. Palm oil consumption represents a saturated fat source relevant for policies aimed at reducing cardiovascular disease burdens.

## Background

The production and human consumption of palm oil, a tropical vegetable oil rich in saturated fats, have risen substantially in recent years, increasing by 40% from 1990 to 2007 in the world's least developed countries [1]. While palm oil production has been used as a tool for economic development in Southeast Asia, controversy has flared over the deleterious environmental effects of its production and the potential that increased consumption damages population health [2,3].

Experts believe that the saturated fat in palm oil worsens cardiovascular health outcomes [2,4]. Experimental evidence confirms that consumption of palm oil increases plasma concentrations of total cholesterol and low-density lipoproteins (LDLs) compared to other more unsaturated vegetable oils [5-7]. Increases in total cholesterol and LDL concentrations in the blood elevate the risk of ischemic heart disease (IHD) [8-12], and randomized controlled trials have clearly shown that replacement of the saturated fats present in usual diets with polyunsaturated fats reduces IHD rates [13]. In Poland, substitution away from saturated fats towards non-hydrogenated rapeseed and soya bean oil was associated with a steep and rapid decline in coronary heart disease mortality between 1990 and 2002, even after adjusting for changes in smoking and fruit and vegetable intake [14,15]. Therefore, although none of these trials consider palm oil explicitly, we hypothesize that increased palm oil consumption, like that observed in many developing countries, raises the risk of IHD. Consistent with this, after per-capita consumption of palm oil increased in Mauritius during a decade of economic growth, a government-led intervention to decrease palm oil use was followed by a substantial drop in mean plasma cholesterol levels [16]. Thus, we believe that potential reductions in cardiovascular mortality are likely related to reductions in palm oil consumption, increasing use of healthier, culturally-acceptable, affordable and available substitute oils, and other changes in the epidemiologic environment (e.g., reductions in smoking).

Ischemic heart disease and cerebrovascular disease -- principally stroke -- are conditions of particular concern for developing countries. With rising household incomes, urbanization, and some success against child mortality, the burden of chronic diseases has increased sharply. In developing countries, health systems long oriented towards infectious diseases now face the challenge of addressing persistent infectious diseases as well as rising chronic disease burdens [4,17,18]. In balancing development, environmental, and health concerns, it is necessary to consider the contribution of increased palm oil consumption to the growing burdens of IHD and stroke in the developing world [4,16-19], particularly in view of both the rapidity and magnitude of the change in IHD mortality in middle-income countries that have decreased their intake of palm oil [16] or butterfat [14,15].

## Methods

### Overview

This study evaluates how palm oil consumption impacts cardiovascular disease mortality rates due to ischemic heart disease (IHD) and stroke. It uses panel data from multiple countries, adjusting for risk factors that have previously been shown to contribute to IHD and stroke deaths. The study covers the period between 1980 and 1997. Each observation describes one year in one country based on data from four publicly available sources that provide information on mortality by cause, domestic consumption of palm oil, domestic consumption of manufactured cigarette products, and other economic, demographic, and nutrition indicators.

### Data

The World Health Organization's (WHO) Mortality Database provides, for each country and year, the numbers of registered deaths for people age ≥ 50 years with underlying causes in two cardiovascular disease (CVD) categories: ischemic heart disease and cerebrovascular disease as coded by the International Classification of Diseases system [20]. Additionally, it provides the population size by country and by year of individuals age ≥ 50 years used to calculate the rates of deaths due to each of the two cardiovascular disease categories. The U.S. Department of Agriculture (USDA) compiles international data on country-level annual total domestic consumption of palm oil for food use [21]. Additionally, it provides information on the annual total domestic consumption of manufactured cigarette products and on domestic food consumption of other major sources of dietary saturated fat: specifically, beef, pork, chicken, coconut oil, milk, eggs and cheese [21]. The World Bank's World Development Indicators (WDI) database provides information on measures of economic development [22]. These measures include per-capita Gross Domestic Product (GDP) expressed in constant 2005 international dollars and country income status (i.e., countries defined as "High-Income Countries" are those ranked in the top 30 nations in terms of per-capita GDP since the 1970s; countries defined as "Developing Countries" are those that have increased their per-capita GDP substantially over this period). The WDI also provides the total population of a country in a given year. All countries in the world were potentially eligible for inclusion in this study's analyses. The only inclusion criterion was that a country must have information on all variables available for at least some years during the period 1980-1997. In the main analyses, there are 234 complete country-year observations from 23 countries. Inclusion of additional covariates in sensitivity analyses reduces the number of country-year observations.

### Model

In the context of economic development, we assess the impact of dietary consumption of palm oil on deaths due to two cardiovascular diseases (IHD and stroke) at the population level, using country-level panel regression analyses. Specifically, we hypothesize that greater palm oil consumption will have negative impacts on population health by increasing cardiovascular disease burdens and mortality of individuals traditionally considered at higher risk for these diseases (those aged 50 and above). Furthermore, we hypothesize that palm oil consumption will be more detrimental in countries undergoing rapid economic development compared to those countries that have been high-income for longer periods of time. The basis for this hypothesis is that increases in palm oil consumption represent an important increase in exposure to saturated fat for the populations of developing countries, relative to high-income countries that have consumed greater quantities of saturated fats for longer periods of time, with fewer fluctuations, and with a major proportion of their saturated fat consumption derived from animal sources. Additionally, health care systems in developing countries are likely less oriented towards chronic diseases care in the study period (1980-1997), a pattern which can lead to greater mortality risk for those with cardiovascular disease even for those receiving medical care in these systems.

The main *outcomes *are mortality rates due to IHD and stroke, computed as the number of deaths among adults aged ≥ 50 from these causes divided by the total population aged ≥ 50 in a given country and year (deaths per 100,000 persons per year). The main *predictors *used are per-capita palm oil consumption and the interaction between the level of palm oil consumption and a country's economic development status (i.e., developing countries and historically high income countries; See Table 1).

**Table 1:** Countries Included in the Analysis by Income Status

*Historically High-Income Countries*
Australia, Canada, Finland, France, Hong Kong, Italy, New Zealand, Netherlands, Norway, Singapore, Spain, Sweden, United States

*Developing Countries*
Brazil, Colombia, Ecuador, Egypt, Greece, Mexico, Peru, Russia, Thailand, Venezuela

Data: World Bank World Development Indicators. "High-Income Countries" are those countries which have, with rare exception, always ranked in the top 30 nations in terms of per capita GDP since the 1970s.

Unlike other countries included in the analysis, Hong Kong's official status is different and has changed over time. However, its economic and administrative organization functioned in ways largely similar to an independent country for the vast majority of the analysis period.

As other factors also impact cardiovascular disease mortality, we adjust for these when assessing the relationship between cardiovascular disease mortality, palm oil consumption, and development. Cigarette smoking is a major risk factor for cardiovascular disease incidence and mortality. We included per-capita consumption of cigarettes, expressed as pieces (i.e., cigarettes) consumed each year per-capita. To account for differential healthcare quality and usage patterns by patients which impact both incidence and mortality from CVDs, we adjust for per-capita GDP as a proxy for health system quality and coverage [23,24]. Per-capita GDP also acts as a proxy for differences in calorie consumption and nutrition over time. Specifically, we include the log of per-capita GDP in the regression model. Finally, as there are temporal shifts in the burden of CVDs, we include indicators for the 1980s to account for other secular trends relative to the 1990s.

### Sensitivity Analyses

Just as consumption of palm oil has changed over the period analyzed, so too have other sources of saturated fat, which could impact the estimated relationship between palm oil and cause-specific mortality. Using the international consumption data compiled by the USDA [21], sensitivity analyses included per-capita consumption of beef, pork, chicken, coconut oil, milk, butter, and cheese. These sources of saturated fat were not included in the main analysis because missing data from their simultaneous inclusion caused too great a loss of sample size. Instead, each saturated fat source was included as an additional covariate to the regression in the main analysis. These sensitivity analyses address the question: Does the estimated relationship between palm oil, a country's level of development and cardiovascular mortality rates attenuate substantially when time-varying consumption of other saturated fats is included? To examine potential collinearity between rises in per-capita palm oil consumption and other sources of dietary saturated fat over time, we regressed per-capita palm oil consumption on these other sources and on per-capita GDP using univariate panel regressions with country fixed effects and robust standard errors.

As the main analysis concerned the potentially differential effect of palm oil consumption in developing countries for whom data were available (Table 1), we assessed whether results were primarily driven by any single country. We repeated the main analysis, estimating the same model but dropping each developing country in turn. We compared these results to those of the main analysis itself.

### Statistical Analyses

We use ordinary least squares panel regressions with country-level fixed effects examining changes in country-level outcomes over time. Because the main outcomes in the models are population averages, we avoid over-representing small nations and under-representing large nations by using inverse variance weights for each country, weighting by a country's average population during the study period. This model specification considers historical variations in palm oil consumption within each country, relying on country fixed effects to mitigate potential sources of time-invariant endogeneity and selection biases that may arise when assessing the mortality effects of palm oil consumption cross-sectionally [25]. All analyses were undertaken using Stata 11 (StataCorp, College Station, Texas, USA).

### Role of Funding Source

The funding sources for this study had no role in the study design; collection, analysis, or interpretation of data; in writing the report; or in the decision to submit the paper for publication. The corresponding author had full access to all data in the study and final responsibility for the decision to submit it for publication.

## Results

**Figure 1:**
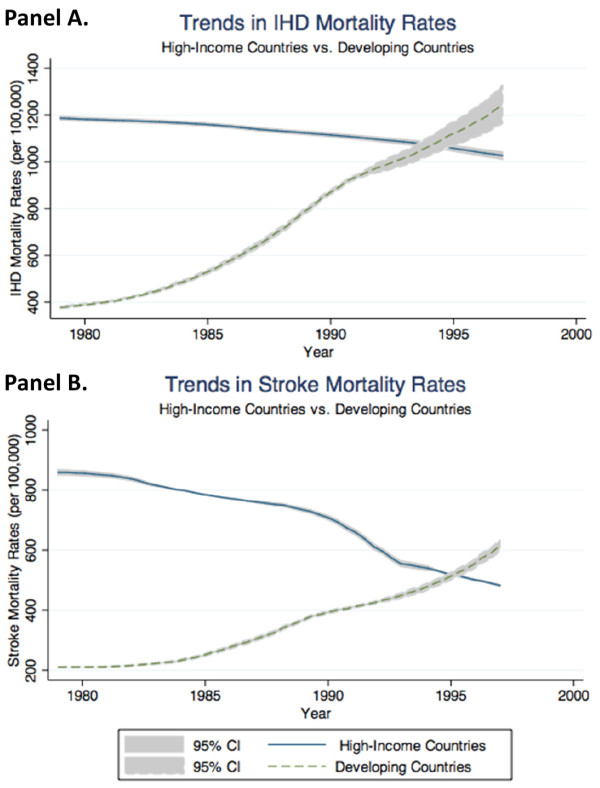
**Trends in Mortality Rates from Ischemic Heart Disease and Stroke in Developing and Historically High-Income Countries**. The figure shows the temporal trends in average ischemic heart disease (IHD) mortality rates (Panel A) and stroke mortality rates (Panel B) disaggregated by country economic development status.

**Figure 2:**
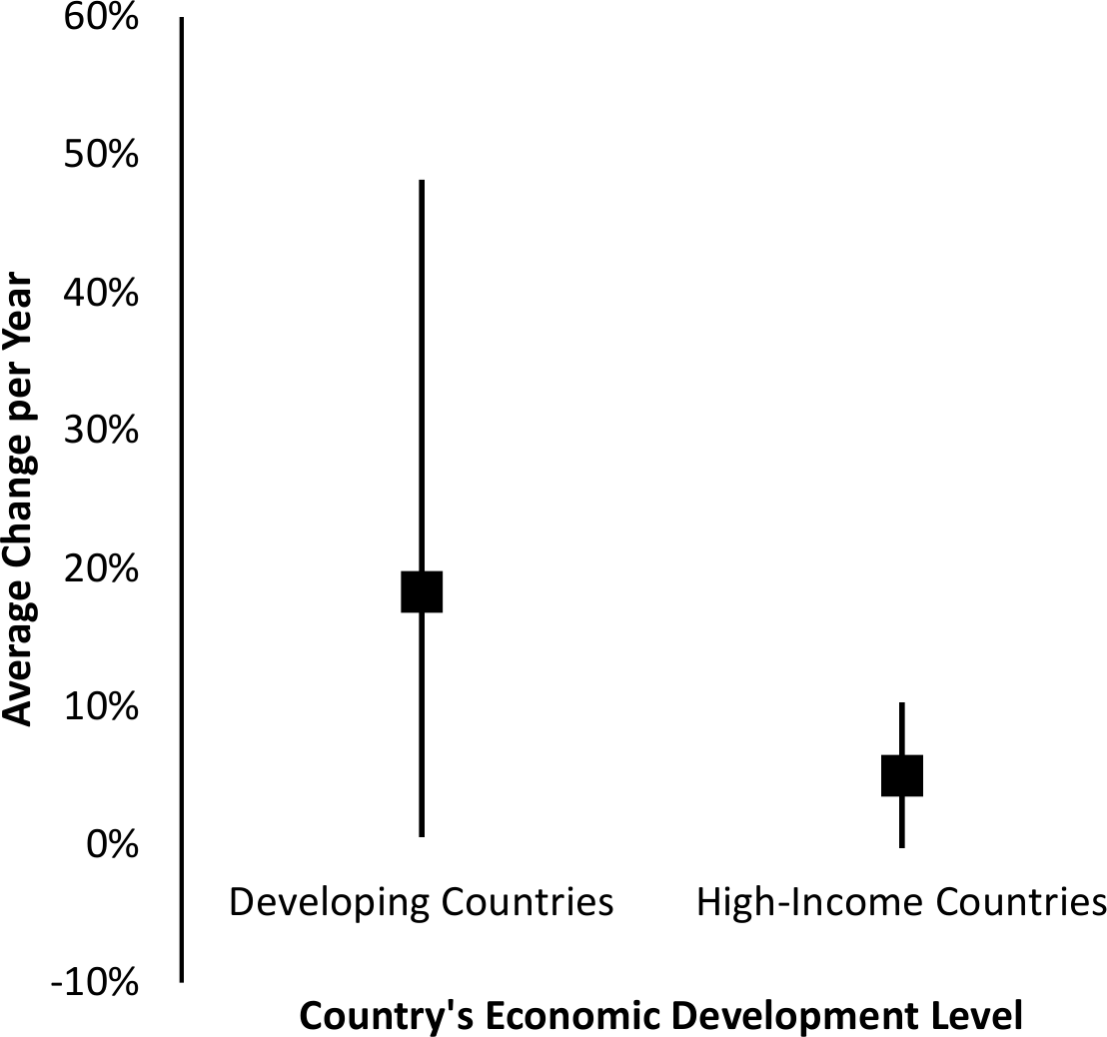
**Changes in Annual Per-Capita Palm Oil Consumption**. The figure shows the mean and interquartile range of increases in per-capita palm oil consumption for developing countries and for historically-high income countries.

Palm oil consumption in developing countries has a significant link to increases in mortality rates for individuals age 50 and above due to ischemic heart disease but not due to stroke, with impacts on health above and beyond those observed in historically high-income countries. Regressions were used to analyze combined yearly data on 10 developing countries and 13 historically high-income countries available from 1980-1997 (Table 1). Compared to historically high-income countries, on average, developing countries had lower mortality rates due to ischemic heart disease (Figure 1, Panel A) and stroke (Figure 1, Panel B) in the 1980s, though these rates increased more rapidly in developing countries, resulting in higher mortality rates from both IHD and stroke in developing countries by the end of the study period. Over this period, developing countries also had significantly lower average annual per-capita consumption of cigarettes and major sources of saturated fat compared to high-income countries with the exception of palm oil and coconut oil and cigarettes compared to high-income countries (Table 2), though they experienced greater growth in their palm-oil consumption than did high-income countries (Figure 2).

**Table 2:** Descriptive Statistics

	Developing Countries		Historically High-Income Countries		Difference** (Developing - High-Income)	
	
	Mean	**Std. Dev**.	Mean	**Std. Dev**.		
	**N = 166**		**N = 244**			
IHD mortality rate (per 100,000)*	753	624	1,144	512	-392	
Stroke mortality rate (per 100,000)*	413	307	723	307	-310	
Per-capita palm oil consumption (kg)	3.5	5.1	4.3	8.2	-0.8	
Per-capita tobacco consumption (pieces)	1,052	857	1,398	687	-345	
Per-capita coconut oil consumption (kg)	1.4	1.4	1.3	1.3	0.1	
Per-capita beef consumption (kg)	19	8.9	24	13	-4.9	
Per-capita milk consumption (kg)	86	59	104	39	-18	
Per-capita butter consumption (kg)	3.5	4.1	4.5	3.6	-1.0	
Per-capita cheese consumption (kg)	3.8	4.4	9.1	4.9	-5.3	
Per-capita pork consumption (kg)	17.2	14	30	11.4	-13	
Per-capita chicken consumption (kg)	9.5	4.7	18	9.5	-8.0	
Per-capita GDP (2005 international $)***	7,914	3,927	23,081	5,230	-15,167	

Data: USDA statistics on country-specific consumption of agricultural commodities (domestic dietary palm oil consumption, domestic consumption of manufactured tobacco products, domestic consumption of other major sources of saturated fat); WHO Mortality Database; World Bank's World Development Indicators, 1980-1997.

* Mortality rates for IHD and stroke are calculated for each country/jurisdiction's population of individuals aged 50 years and older.

** All differences significant (t-test) at p < 0.05 except per-capita palm oil consumption and per-capita coconut oil consumption

*** Per-capita GDP calculated using purchasing power parity (PPP)

We report the number of observations (N) at the country-year level.

There are 10 "developing economies" (countries that have grown in wealth since the 1970s) and 13 "high-income countries" (countries that have been historically wealthy since the 1970s) in the data set.

Palm oil consumption in developing countries incurs greater negative health effects than in historically high-income countries, even after adjustment for other major risk factors for deaths from IHD and stroke, including cigarette consumption, economic growth, and secular trends over time. In developing countries, for every additional kilogram of palm oil consumed annually per capita, the IHD mortality rate increased by 68 deaths per 100,000 (95% CI [21-115]); and the mortality rate for stroke increased by 19 deaths per 100,000 (95% CI [-12-49]), but the increase in stroke deaths was not statistically significant (Table 3). For high-income countries, changes in the mortality rates of both IHD and stroke deaths with every additional kilogram of palm oil consumed annually per-capita are substantially smaller for both IHD and stroke and not significant for stroke at 17 deaths per 100,000 (95% CI [5.3-28]) and 5.1 deaths per 100,000 (95% CI [-1.2-11]), respectively (Table 3). The significant and larger increases in IHD deaths due to palm oil consumption and lack of a similar significant effect for stroke are consistent with the underlying causes of IHD and stroke, respectively, as IHD is primarily due to atherosclerosis, a process influenced strongly by plasma cholesterol and LDL levels [4,12,13]. However, many strokes are of hemorrhagic origin with a major causal antecedent being hypertension. Palm oil and other saturated fats have not been shown to increase blood pressure, and therefore should have a weaker link to stroke deaths.

**Table 3:** Palm Oil Consumption and IHD and Stroke Mortality Rates

	IHD Mortality Rate (per 100,000 adults age ≥ 50 years)		Stroke Mortality Rate (per 100,000 adults age ≥ 50 years)	
Palm oil consumption (kg per-capita)	17.0	[5 - 29]	5.1	[-1.2 - 11]
Additional effect of palm oil in developing countries (kg per-capita)	51	[3.9 - 98]	13	[-18 - 45]
Cigarette consumption (pieces per-capita)	0.05	[-0.15 - 0.25]	-0.08	[-0.19 - 0.02]
Log per-capita GDP (%)	-2,132	[-3,130 - -1,133]	-930	[-1,307 - -552]
1980s (compared to 1990s)	-166	[-349 - 17]	-70	[-135 - -4.1]
R-squared	0.58		0.54	
Observations (country-years)	234		234	
Number of countries	23		23	

Regressions use robust standard errors clustered by country

Higher cigarette consumption also increases the proportion of deaths from cardiovascular disease. In the analysis, for each additional cigarette per-capita smoked per year, there are an estimated additional 0.05 IHD deaths per 100,000 (95% CI [-0.15-0.25]), though for stroke, the estimate is -0.08 deaths per 100,000 (95% CI [-0.19-0.02]). Just as with palm oil consumption, smoking's stronger impact, while not significant, appears to be on changes in IHD deaths, consistent with the known strong connection in developed countries between cigarette smoking and IHD, but with a neutral to slightly negative correlation with hypertension [26,27].

Analyses examined whether estimates of palm oil consumption's link to differentially higher IHD mortality in developing countries could be explained by simultaneous increases in consumption of other major sources of saturated fat. When per-capita consumption of beef, coconut oil, milk, pork, or chicken were each individually included in the regression, despite a loss of sample size (n between 153 and 217 country-years), the estimated effect of palm oil in these countries remained significant at or above 55 deaths per 100,000 (Table 4). For butter or cheese, where substantial sample size was lost (n = 177 country-years), the effect of palm oil consumption on IHD mortality lost significance, but its estimated effect remained in the same range (58 to 66 IHD deaths per 100,000 range). Per-capita palm oil consumption itself was significantly related to greater per-capita consumption of pork and to higher per-capita GDP but not to other major sources of dietary saturated fat intake (Table 5).

**Table 4:** The Relationship between Palm Oil Consumption and IHD Mortality Rates in High Income and Developing Countries Remains Unchanged when Adjusting for Other Sources of Dietary Saturated Fat

Dependent variable in all regressions: IHD mortality	Adjusting for Beef Consumption	Adjusting for Coconut Oil Consumption	Adjusting for Milk Consumption	Adjusting for Butter Consumption	Adjusting for Cheese Consumption	Adjusting for Pork Consumption	Adjusting for Chicken Consumption
Palm oil consumption (kg per-capita)	18.9	3.3	-3.4	-1.2	1.7	19	19
	[7.7 - 30]	[-1.4 - 7.9]	[-39 - 32]	[-43 - 41]	[-40 - 43]	[8.0 - 30]	[8.4 - 30]

Additional effect of palm oil in developing countries (kg per-capita)	52	149	70	59	65	41	72
	[26 - 78]	[93 - 205]	[19 - 120]	[-58 - 175]	[-58 - 188]	[10 - 72]	[18 - 126]

Cigarette consumption (pieces per-capita)	-0.00	0.26	0.01	0.05	0.07	-0.03	0.11
	[-0.29 - 0.29]	[0.15 - 0.37]	[-0.18 - 0.21]	[-0.20 - 0.29]	[-0.15 - 0.28]	[-0.23 - 0.18]	[-0.26 - 0.48]

Log per-capita GDP (%)	-2,259	-728	-2,262	-2,116	-2,469	-2,370	-2,191
	[-3,201 - -1,317]	[-1,061 - -395]	[-2,830 - -1,695]	[-2,851 - -1,381]	[-3,160 - -1,779]	[-3,271 - -1,469]	[-3,002 - -1,381]

1980s (compared to 1990s)	-176	6.7	-171	-133	-152	-162	-154
	[-348 - -4.7]	[-68 - 82]	[-341 - -1.2]	[-304 - 38]	[-328 - 23]	[-353 - 28]	[-425 - 118]

Beef consumption (kg per-capita)	6.6						
	[-17 - 30]						
Coconut oil consumption (kg per-capita)		21					
		[-35 - 77]					
Milk consumption (kg per-capita)			4.8				
			[-0.7 - 10]				
Butter consumption (kg per-capita)				-50			
				[-119 - 19]			
Cheese consumption (kg per-capita)					32		
					[-15 - 79]		
Pork consumption (kg per-capita)						9.6	
						[-10 - 29]	
Chicken consumption (kg per-capita)							6.3
							[-30 - 43]

Constant	23,221	7,942	23,066	22,180	25,213	24,411	22,299
	[13,631 - 32,811]	[4,505 - 11,379]	[17,424 - 28,708]	[14,900 - 29,460]	[18,243 - 32,183]	[15,454 - 33,368]	[13,483 - 31,115]

							

Observations	217	153	181	177	177	209	202
R-squared	0.60	0.89	0.70	0.64	0.64	0.61	0.59
Number of countries	22	13	20	19	19	21	20

**Table 5:** Univariate Relationships of Palm Oil Consumption and Other Sources of Dietary Saturated Fat and Per-capita GDP

	Palm oil consumption (kg per-capita)	P-value	95% Confidence Interval
Per-capita coconut oil consumption (kg)	-1.58	0.09	[-3.44 - 0.28]
Per-capita beef consumption (kg)	-0.06	0.42	[-0.19 - 0.08]
Per-capita milk consumption (kg)	0.00	0.66	[-0.01 - 0.01]
Per-capita butter consumption (kg)	0.02	0.77	[-0.13 - 0.18]
Per-capita cheese consumption (kg)	0.14	0.44	[-0.22 - 0.50]
Per-capita pork consumption (kg)	0.08	0.002	[0.03 - 0.13]
Per-capita chicken consumption (kg)	0.03	0.70	[-0.13 - 0.19]
Per-capita GDP (2005 international $)*	8.61	0.01	[2.01 - 15.20]

* Per-capita GDP is logged transformed so the coefficient can be interpreted as increases in per-capita consumption of palm oil for each percent increase in per-capita GDP.

In addition to adjusting for country size, we ensured that no single growing economy country overly influenced the results by removing each of these 10 growing economy countries individually and performing the analysis 10 times. All results are substantially similar to the main analysis described above. The estimated effect of palm oil consumption on IHD-specific mortality rates and the differentially higher effect of palm oil consumption in developing countries were stable across countries, though significance of the differential effect in developing countries was attenuated by the removal of Colombia, Egypt, or Thailand (Table 6).

**Table 6:** Sensitivity Analysis: Impact of Palm Oil Consumption on IHD Mortality, with Growing Economies Removed One at a Time

Dependent variable: IHD mortality	Removed Brazil	Removed Colombia	Removed Ecuador	Removed Egypt	Removed Greece	Removed Mexico	Removed Peru	Removed Russia	Removed Thailand	Removed Venezuela
Palm oil consumption (kg per-capita)	16	17	17	18	17	18	17	5	17	17
95% CI	[4.5 - 28]	[5.1 - 29]	[5.6 - 29]	[6.1 - 29]	[5.2 - 29]	[6.2 - 29]	[5.5 - 29]	[-3.9 - 14]	[6.0 - 29]	[5.4 - 29]

Additional effect of palm oil in developing country (kg per-capita)	50	50	69	46	51	52	51	63	38	49
95% CI	[3.5 - 97]	[-62 - 161]	[23 - 116]	[-7.7 - 99]	[3.5 - 98]	[3.4 - 100]	[4.3 - 99]	[28 - 99]	[-3.8 - 80]	[1.0 - 96]

Observations (country-years)	225	227	226	231	229	223	230	225	226	228
R-squared	0.65	0.58	0.59	0.59	0.58	0.59	0.58	0.45	0.59	0.58
Number of countries	22	22	22	22	22	22	22	22	22	22

Robust standard errors clustered by country. Econometric specification is identical to that of Table 3. Regression analysis was performed eleven times, with a single developing country (listed in the table heading) removed from the sample to test whether any one country was pivotal in the results reported in Table 3. For clarity of exposition, however, only the coefficient of interest is presented.

## Discussion

In developing countries, increases in palm oil consumption are associated with higher mortality due to ischemic heart disease (IHD) to a greater degree than in countries that have been historically wealthy. These increases occur above and beyond those caused by smoking and other important economic, demographic, and nutritional trends.

Global palm oil consumption represents an important health policy challenge. The consumption of palm oil overtook soybean oil consumption globally in 2003, and the increasing price competitiveness of palm oil relative to soybean oil has resulted in palm oil's status as the dominant oil on the global market [28]. The world market for palm oil is forecast to surpass 100 million metric tons by 2015, fueled primarily by demand in growing economies such as China and India [29]. Their future palm oil consumption could potentially increase cardiovascular disease-specific mortality.

National and international organizations should consider health as part of their evaluations of the potential benefits and harms of palm oil production and consumption, weighing these against relevant alternatives in light of a growing body of evidence regarding the disproportionate health harms of saturated fat in the context of rapidly developing countries. Currently, the World Bank's Draft Framework for Engagement in the Palm Oil Sector cites "poverty reduction" as a key rationale for supporting palm oil production, citing "negative environmental and social impacts" but not health concerns as potential drawbacks of the strategy [30]. The case of palm oil represents a more general problem that programs focused on economic development and those focused on health are often siloed. As part of the discussion around the United Nation's High-level Meeting on Non-communicable Disease Prevention and Control in September 2011, recent calls have included the development of "healthy crops" and policies that encourage their sustainability [31] and the deeper involvement of the World Health Organization in food and agricultural production to support these efforts [32].

Policies that curtail palm oil consumption in rapidly developing countries require careful consideration. One policy now in use to lower CVD risk in developed countries has been to reduce the use of hydrogenated oils, which contain the trans-fats that raise LDL in a similar manner to that of saturated fats. However, in many countries, this has led to substitution of palm oil for trans-fats, which may undermine the health goals of these efforts. Nutritional and environmental concerns associated with palm oil production and consumption may prompt a call for measures to increase the relative price of palm oil. Benefits of such a policy must be weighed against what fat substitutes would be made, especially by poorer individuals, if the prices of both trans-fats and saturated fats were targeted via tax policies.

Our findings are consistent with the extensive literature documenting the link between the consumption of saturated fats (such as palm and coconut oil, as well as animal fats) on plasma total cholesterol and LDL cholesterol and ischemic heart disease [2,4-6,8-13]. National and international organizations including the World Health Organization and U.S. Departments of Agriculture and of Health and Human Services recommend consuming fewer saturated fats as opposed to monounsaturated or polyunsaturated fats to reduce the prevalence of cardiovascular diseases [33,34].

Our findings are also consistent with the previous results that show that high serum LDL levels cause predominantly IHD and some strokes through blockage (thrombosis) in vessels narrowed by atherosclerosis, whereas LDL has no important relationship to hypertension, which can lead to many strokes from hemorrhages of the cerebral blood vessels. Therefore, our finding of a weaker relationship between palm oil and stroke than IHD is consistent with the expected role of higher LDL levels.

The relationship between increased palm oil consumption in developing countries and IHD mortality is consonant with the previously mentioned link between saturated fat consumption and IHD mortality. The weaker relationship between palm oil consumption and IHD mortality in historically high-income countries could be due to a variety of reasons. In many historically high-income countries screening for cardiovascular diseases is more common, the use of drugs such as statins for both primary and secondary prevention is more widespread, and, in general, the quality of hospital care and availability of advanced lifesaving technologies may be better. Additionally, in many historically high-income countries, the proportion of diets that comes from saturated fats is more stable. Also, the lower marginal increases in CVD deaths from increased palm oil consumption in historically high-income countries may be due to the fact that at baseline palm oil consumption levels were already relatively high and saturated fats from animal products remained the major sources of saturated fat for most people's diets.

There are several limitations to this study. First, major palm oil producing and consuming countries including Indonesia and Malaysia were not represented in the present analysis because complete data on other characteristics including cause-specific mortality and cigarette consumption were not available for the time period considered. The relationship of palm oil consumption and health in these settings is of great interest and should be explored when such data become available. Second, cigarette consumption per-capita does not have a statistically significant relationship with cardiovascular mortality outcomes in our analysis. This could potentially be due to collinearity with other variables in the regression. Of note, cigarette smoking is expressed as additional pieces (e.g., cigarettes) consumed per-capita each year. While pack consumption or pack-days are often used, the quantity of cigarettes in a pack changed within countries and over time in our multi-country analysis prompting us to report outcomes in per-cigarette terms to avoid potential confusion as to the definition of packs when the results are interpreted. For example, a common pack size in Australia is 25 although 20-40 cigarette packs are sold. In Canada, it is 20-25, and in Malaysia, a pack can have as few as 14 cigarettes. In fact, in some developing countries, stores often sell individual cigarettes. Third, the use of country-level data in this ecologic study may mask individual variability in the consumption of palm oil and its real impact on health. Importantly, our study employs an econometric model that does not explicitly adjust for all factors that affect CVD deaths. However, by employing country fixed effects that control for time-invariant confounders we partially address omitted variable bias. Our regression results may have a causal interpretation if we assume that unaccounted IHD and stroke risk factors remain constant within each country throughout the study period and no selection biases exist. We also make no cross-sectional comparisons, avoiding selection issues associated with non-randomized samples. However, if palm oil consumption is correlated with other time-varying IHD and stroke risk factors then our analysis cannot separately disentangle the causal impact of palm oil consumption on IHD and stroke from the effects of these other factors. Despite our adjustment for cigarette use and per-capita GDP, the possibility of other unmeasured changes contributing to the effect is real as developing countries have seen nutritional transitions that include increased obesity rates. Our sensitivity analyses suggest that the effect we have estimated for IHD in developing countries is fairly robust with respect to changing patterns of consumption of other major sources of saturated fat, though other factors not included may still play an important role. Nonetheless, ecological estimates that are consistent with smaller randomized trials examining intermediate outcomes and known biological mechanisms contribute significantly to the evidence on important issues like the link between palm oil consumption and CVD mortality.

## Conclusions

This study is the first to document a significant relationship between increased palm oil consumption and higher IHD mortality rates at a population level in multiple countries. As the evidence base linking palm oil consumption and population health grows, decision makers should consider policies that focus on consumption of both plant and animal sources of saturated fat to address rapidly rising ischemic heart disease mortality in developing countries.

## List of abbreviations

CI: Confidence Interval; CVD: Cardiovascular Disease; GDP: Gross Domestic Product; ICD: International Classification of Disease; IHD: Ischemic Heart Disease; KG: kilograms; LDL: Low Density Lipoproteins; PPP: Purchasing Power Parity; St. Dev.: Standard Deviation; USDA: United States Department of Agriculture; WDI: World Development Indicators; WHO: World Health Organization.

## Conflicts of interests

The authors declare that they have no competing interests.

## Authors' contributions

BKC participated in the conception and design of the study, collected data, performed statistical analyses, drafted sections of the original manuscript, and provided critical comments and revisions. BS participated in the conception of the study and provided critical comments and revisions. JWF participated in the conception of the study and provided critical comments and revisions. JGF participated in the conception and design of the study, collected data, drafted sections of the original manuscript, and provided critical comments and revisions. All authors read and approved the final manuscript.

## References

[B1] FAOSTAT Food Balance Sheets http://faostat.fao.org/site/368/default.aspx#ancor

[B2] Brown E Jacobson M Cruel Oil: How Palm Oil Harms Health, Rainforest & Wildlife 2005 Center for Science in the Public Interest

[B3] Sheil M Casson A Meijaard E van Noordwijk M Gaskell J The Impacts and Opportunities of Oil Palm in Southeast Asia 2009 Jakarta: Center for International Forestry Research

[B4] Fuster B Kelly B Promoting Cardiovascular Health in the Developing World: A Critical Challenge to Achieve Global Health 2010 Washington, D.C.: The National Academies Press 20945571

[B5] Katan M Zock P Mensink R Effects of Fats and Fatty Acids on Blood Lipids in Humans: An Overview American Journal of Clinical Nutrition 1994 60 1017S 1022S 7977143 10.1093/ajcn/60.6.1017S

[B6] Nicolosi R Rogers E Regulation of Plasma Lipoprotein Levels by Dietary Triglycerides Enriched with Different Fatty Acids Medicine & Science in Sports & Exercise 1997 29 1422 1429 10.1097/00005768-199711000-00006 9372477

[B7] Vega-Lopez S Ausman L Jalbert S Erkkila A Lichtenstein A Palm and Partially Hydrogenated Soybean Oils Adversely Alter Lipoprotein Profiles Compared with Soybean and Canola Oils in Moderately Hyperlipidemic Subjects American Journal of Clinical Nutrition 2006 84 54 62 16825681 10.1093/ajcn/84.1.54

[B8] Hu F Willett W Optimal Diets for Prevention of Coronary Heart Disease Journal of the American Medical Association 2002 288 2569 2578 10.1001/jama.288.20.2569 12444864

[B9] Kannel W Castelli W Gordon T McNamara P Serum Cholesterol, Lipoproteins, and the Risk of Coronary Heart Disease Annals of Internal Medicine 1971 74 1 12 5539274 10.7326/0003-4819-74-1-1

[B10] Manninen V Elo M Frick M Haapa K Heinonen O Heinsalmi P Helo P Huttunen J Kaitaniemi P Koskinen P Lipid Alterations and Decline in the Incidence of Coronary Heart Disease in the Helsinki Heart Study Journal of the American Medical Association 1988 260 641 651 10.1001/jama.1988.03410050061031 3164788

[B11] Menotti A Keys A Blackburn H Kromhout D Karvonen M Nissinen A Pekkanen J Punsar S Fidanza F Giampaoli S Comparison of Multivariate Predictive Power of Major Risk Factors for Coronary Heart Diseases in Different Countries: Results from Eight Nations of the Seven Countries Study, 25-Year Follow-Up Journal of Cardiovascular Risk 1996 3 69 10.1097/00043798-199602000-00010 8783033

[B12] Wilson P D'Agostino R Levy D Belanger A Silbershatz H Kannel W Prediction of Coronary Heart Disease Using Risk Factor Categories Circulation 1998 97 1837 1847 9603539 10.1161/01.cir.97.18.1837

[B13] Mozaffarian D Micha R Wallace S Effects on Coronary Heart Disease of Increasing Polyunsaturated Fat in Place of Saturated Fat: A Systematic Review and Meta-Analysis of Randomized Controlled Trials PLoS Med 2010 7 e1000252 10.1371/journal.pmed.1000252 20351774 PMC2843598

[B14] Zatonski WA McMichael AJ Powles JW Ecological study of reasons for sharp decline in mortality from ischaemic heart disease in Poland since 1991 British Medical Journal 1998 316 1047 1051 10.1136/bmj.316.7137.1047 9552904 PMC28506

[B15] Zatonski WA Willett W Changes in dietary fat and declining coronary heart disease in Poland: population based study British Medical Journal 2005 331 187 188 10.1136/bmj.331.7510.187 16037448 PMC1179759

[B16] Uusitalo U Feskens E Tuomilehto J Dowse G Haw U Fareed D Hemraj F Gareeboo H Alberti K Zimmet P Fall in Total Cholesterol Concentration over Five Years in Association with Changes in Fatty Acid Composition of Cooking Oil in Mauritius: Cross Sectional Survey British Medical Journal 1996 313 1044 1046 10.1136/bmj.313.7064.1044 8898594 PMC2352358

[B17] Strong K Mathers C Leeder S Beaglehole R Preventing chronic diseases: how many lives can we save? The Lancet 2005 366 1578 1582 10.1016/S0140-6736(05)67341-2 16257345

[B18] Mathers CD Loncar D Projections of Global Mortality and Burden of Disease from 2002 to 2030 PLoS Med 2006 3 e442 10.1371/journal.pmed.0030442 17132052 PMC1664601

[B19] World Health Organization Preventing Chronic Diseases: A Vital Investment 2005 Geneva: World Health Organization

[B20] WHO Mortality Database http://www.who.int/whosis/mort/download/en/index.html

[B21] Index Mundi -- International commodity and consumption information as compiled by the United States Department of Agriculture http://www.indexmundi.com/

[B22] The World Bank World Bank: World Development Indicators 2008 Book World Bank: World Development Indicators

[B23] Goldhaber-Fiebert JD Lipsitch M Mahal A Zaslavsky AM Salomon JA Quantifying Child mortality reductions related to measles vaccination PloS One 2010 5 e13842 10.1371/journal.pone.0013842 21079809 PMC2973966

[B24] Anand S Bärnighausen T Health Workers and Vaccination Coverage in Developing Countries: An Econometric Analysis The Lancet 2007 369 1277 1285 10.1016/S0140-6736(07)60599-6 17434403

[B25] Greene WH Econometric Analysis 2002 Fifth Prentice Hall

[B26] Friedman G Klatsky A Siegelaub A Alcohol, tobacco, and hypertension Hypertension 1982 4 143 10.1161/01.hyp.4.5_pt_2.iii143 7049929

[B27] U.S. Department of Health and Human Services The Health Consequences of Smoking: A Report of the Surgeon General 1982 Rockville, MD: Public Health Service, Office of Smoking and Health

[B28] Foreign Agricultural Service Palm Oil Continues to Dominate Global Consumption in 2006/07 USDA Circular Series 2006 FOP 6-06

[B29] Why global palm oil market is bullish and expanding http://www.commodityonline.com/news/Why-global-palm-oil-market-is-bullish-and-expanding-25787-3-1.html

[B30] Framework for Engagement in the Palm Oil Sector http://www.ifc.org/ifcext/agriconsultation.nsf/AttachmentsByTitle/Draft+Framework+Paper+for+consultations/$FILE/WBG_Framework_for_Palm_Oil-DRAFT+FOR+CONSULTATION.pdf

[B31] Bringing Agriculture to the Table http://www.thechicagocouncil.org/UserFiles/File/GlobalAgDevelopment/Report/Bringing_Agriculture_To_The_Table.pdf

[B32] Yach D Nutritional change is not a simple answer to non-communicable diseases Bmj 2011 343 d5097 10.1136/bmj.d5097 21835872

[B33] King J Appel L Caballero B Clydesdale F Kris-Etherton P Nicklas T United States Department of Health and Human Services: Dietary Guidelines for Americans 2005

[B34] Swinburn B Baba N Belhadj M Deurenberg-Yap M Djazayery A Forrester T Galuska D Herman S James W M'Buyamba Kabangu J Diet, Nutrition and the Prevention of Chronic Diseases: Report of a Joint WHO/FAO Expert Consultation 2002 Geneva: World Health Organization

